# Medical Opinion and Movement

**Published:** 1907-02-16

**Authors:** 


### Medical Opinion and Movement.

It is reported that for some months past a special ward has been set apart at the Finsen Light Cure Institute, Copenhagen, for the treatment of diseases of the heart and other organic maladies. Finsen light baths are given to the patients, and a certain amount of success is claimed for the treatment. Professor Finsen is said to have had in view a further extension of the therapeutic effects of his light treatment before his death. No details are as yet to hand as to the actual methods pursued or the results obtained. One would hardly expect that the treatment would have anything but a palliative effect upon those morbid conditions which owe their origin to some definite organic lesion of an internal organ, such as valvular heart disease; but if the treatment can be shown to effect as much as medicinal or other palliative measures, it will deserve the attention of the profession, and will be entitled to rank among the therapeutic means at our command. It is, however, idle to speculate as to the nature of the action of the light upon the condition of the patient until we have received further data from Copenhagen.

Surgeons are realising more and more the importance of attention to the smallest details, both in operative procedure and also in the general treatment of the patient, in order to secure the most favourable results. This remark applies most especially to abdominal surgery. Mr. Charles A. Morton, F.R.C.S., Professor of Surgery in University College, Bristol, gives several useful hints in an address on the subject. In suppurative conditions of the abdominal cavity the patient should be placed in "Fowler's position"; that is, in the half-sitting position, with the chest propped up by pillows or a bed-rest. This should not be left to the nurse to arrange, unless it is known that she is thoroughly competent by previous experience to carry it out. The patient naturally tends to slip down in the bed away from the support; in order to prevent this, a sling of cloth may be carried round both sides of the body and the buttocks and perineum, and each end attached to the head of the bed. An alternative plan is to place a wooden inclined plane, covered by a pillow, under the bent knees, and attach it to the head of the bed by adjustable cords. Tilting up the lower end of the bed is quite inadequate for the purpose. In most cases this position should be maintained for twenty-four to forty-eight hours, when adhesions should be sufficiently formed to prevent spread of infection to the upper part of the abdomen. Another important detail is to prevent the patient from contracting his sewn-up abdominal muscles. The smallest voluntary effort to raise the shoulders is accompanied by a strong contraction of the abdominal muscles. The patient should therefore be instructed to keep himself quite passive during any attempt to raise him in the bed.

In the address referred to, Mr. C. Morton lays claim to the right of the surgeon, who is called in to operate upon a patient, to direct the after-treatment of the case. We should hardly have thought that such a claim would be contested. But it is evident from what Mr. Morton has to say on the subject that he has encountered some difficulty in making good his claim. It is not fair to regard a surgeon in the light of a mechanic who is called in simply to execute a piece of manual work. When the surgeon operates he takes a very large share in the responsibility of the case, and his interests and reputation are involved in the subsequent result. Much may depend for that result upon the way in which the case is treated after the operation, and it is only right that he should be consulted and his wishes followed in regard to that treatment. It is not to be expected that the medical attendant who has had charge of the case up to the time of operation will retire altogether in favour of the operating surgeon, unless the patient is removed from his previous surroundings and placed under the care of the surgeon in a nursing home. In private practice, when the patient is operated upon at home, the medical attendant naturally remains in charge of the case; but he should be ready, and we cannot but think that he is usually only too ready to receive advice from the surgeon, and, in fact, to carry out the treatment of the case in accordance with his instructions. Different surgeons evince a varying amount of interest in the subsequent course of their cases, but the majority are willing to make further visits when necessary, independently of any fees; and we believe that such assistance is generally appreciated cordially by the general practitioner.

In a previous issue we referred to Dr. H. D. McCulloch's experiments with $x$-rays, by which he was able to show that their action upon local manifestations of tuberculosis was accompanied by a rise in the opsonic index of the patient. We have since received a communication from him in which he draws our attention to "points of vital importance" in his paper, which we passed over in referring to it. These points to which the author refers are rather questions of hypothesis than of fact. Dr. McCulloch devotes the first part of his paper to a very able consideration of the processes which take place in the body, by which an individual is rendered more or less immune to a given disease. He draws a very plausible and graphic picture of the probable course of events. He can, however, hardly lay claim to originality for the picture. It is the "working hypothesis" of bacteriologists of the present day, and on that account chiefly we did not think any special reference to this part of the paper necessary. We recognise that it is unsatisfactory for an author to find only one half of his ideas reported or represented, but in this case we confined ourselves to the practical observation that the action of $x$-rays was accompanied by changes in the opsonic index of a tubercular patient and indicated the manner in which Dr. McCulloch suggested these changes were brought about.

